# *Allium victorialis* L. Extracts Promote Activity of FXR to Ameliorate Alcoholic Liver Disease: Targeting Liver Lipid Deposition and Inflammation

**DOI:** 10.3389/fphar.2021.738689

**Published:** 2021-10-08

**Authors:** Zhen-Yu Cui, Xin Han, Yu-Chen Jiang, Jia-Yi Dou, Kun-Chen Yao, Zhong-He Hu, Ming-Hui Yuan, Xiao-Xue Bao, Mei-Jie Zhou, Yue Liu, Li-Hua Lian, Xian Zhang, Ji-Xing Nan, Yan-Ling Wu

**Affiliations:** ^1^ Key Laboratory for Traditional Chinese Korean Medicine of Jilin Province, College of Pharmacy, Yanbian University, Yanji, China; ^2^ Chinese Medicine Processing Centre, College of Pharmacy, Zhejiang Chinese Medical University, Hangzhou, China; ^3^ Agricultural College, Yanbian University, Yanji, China; ^4^ Clinical Research Center, Affiliated Hospital of Yanbian University, Yanji, China

**Keywords:** *Allium victorialis* L, alcoholic liver disease, lipid deposition, inflammation, farnesoid X receptor

## Abstract

*Allium victorialis L.* (AVL) is a traditional medicinal plant recorded in the Compendium of Materia Medica (the Ming Dynasty). In general, it is used for hemostasis, analgesia, anti-inflammation, antioxidation, and to especially facilitate hepatoprotective effect. In recent years, it has received more and more attention due to its special nutritional and medicinal value. The present study investigates the effect and potential mechanism of AVL against alcoholic liver disease (ALD). C57BL/6 mice were fed Lieber–DeCarli liquid diet containing 5% ethanol plus a single ethanol gavage (5 g/kg), and followed up with the administration of AVL or silymarin. AML12 cells were stimulated with ethanol and incubated with AVL. AVL significantly reduced serum transaminase and triglycerides in the liver and attenuated histopathological changes caused by ethanol. AVL significantly inhibited SREBP1 and its target genes, regulated lipin 1/2, increased PPARα and its target genes, and decreased PPARγ expression caused by ethanol. In addition, AVL significantly enhanced FXR, LXRs, Sirt1, and AMPK expressions compared with the EtOH group. AVL also inhibited inflammatory factors, NLRP3, and F4/80 and MPO, macrophage and neutrophil markers. *In vitro*, AVL significantly reduced lipid droplets, lipid metabolism enzymes, and inflammatory factors depending on FXR activation. AVL could ameliorate alcoholic steatohepatitis, lipid deposition and inflammation in ALD by targeting FXR activation.

## Introduction

Alcohol consumption is highly addictive and associated with social, economic, and multifarious health problems (Galicia-Moreno and Gutiérrez-Reyes, 2014). Although moderate drinking is beneficial to physical and mental health, such as prevention of heart disease, hypertension, and diabetes, excessive and prolonged drinking leads to alcoholic liver disease (ALD) (Berger et al., 1999; Sacco et al., 1999). ALD is the main pathogenic factor contributing to morbidity of liver diseases worldwide. Generally, alcohol is first metabolized to acetaldehyde by alcohol dehydrogenase and partly metabolized by cytochrome P4502E1 (CYP2E1), catalase in hepatocyte microsomes and peroxisomes (Liu, 2014). This process would synthesize fatty acids, accumulate triglycerides (TGs) and further lead to fatty liver disease. Simple fatty liver is the early stage in ALD progression and would subsequently lead to steatosis, alcoholic hepatitis, fibrosis, and even cirrhosis with continuous consumption of excessive amounts of alcohol (Shearn et al., 2013; Addolorato et al., 2016). So far, there has been no specific drug to prevent or treat fatty liver. And it is still critical to develop an effective therapeutic drug for ALD, which not only prevents progression and reduces fat deposition but also accelerates regeneration and stability of hepatocytes and promotes liver activity.

Hepatic steatosis is part of the early adaptive response of the liver against some chronic stimulus. Inflammation might occur before hepatic steatosis and subsequently lead to steatosis. During the progression of steatosis, nutrients and their metabolites can also induce the secretion of adipokines and inflammatory factors by adipocytes, macrophages, and other cells, which is called metabolically triggered inflammation (Hotamisligil, 2006). ALD in patients is often accompanied by dyslipidemia, elevation of free fatty acids, and related lipotoxicity, insulin resistance and intestinal endotoxin, which contribute to stimulate and maintain the production and release of proinflammatory cytokines. Thus, inflammation is involved in the development of ALD.

Current researches have indicated that alcohol and its metabolites play critical roles in ALD development through hepatotoxicity, oxidative stress, lipid peroxidation, and ethanol metabolic enzyme system. Hepatosteatosis is the early abnormality in the pathogenesis of ALD due to chronic alcohol abuse and metabolic syndrome. Chronic alcoholism can effect fat synthesis and inhibit fatty acid oxidation, including upregulating SREBP1 and PPARγ by targeting key transcriptional genes associated with metabolic processes (Esfandiari et al., 2005; Esfandiari et al., 2007). Chronic alcohol consumption increased expressions of SREBP1 and PPARγ that is related to the activation of NAD-dependent deacetylase Sirtuis (Sirt) and adenosine 5'-monophosphate (AMP)–activated protein kinase (AMPK) (Lieber et al., 2008). Moreover, chronic alcohol intake could break the mutual effect between farnesoid X receptor (FXR; NR1H4) and retinoid X receptor α (RXRα; NR2B1) via acetylation and inactivation of FXR (Wu et al., 2014). FXR is a nuclear hormone receptor (NR), which is involved in the regulation of bile acid homeostasis and further in liver disease via the gut–liver axis (Forman et al., 1995; Seol et al., 1995). A previous study had shown that chronic alcohol consumption impaired FXR activity, and activated FXR attenuates hepatic liver injury, steatosis, and cholestasis induced by ETOH (Wu et al., 2014). FXR activation decreases TG levels to regulate lipogenesis through the downregulation of SREBP1 or upregulation of PPARα, mediating fatty acid oxidation (Pineda Torra et al., 2003; Watanabe et al., 2004; Ding et al., 2014). Thus, the present study focuses on the important role FXR plays in the development of ALD caused by Lieber–DeCarli ethanol liquid diet and contributes to alterations of lipid deposition and inflammation through a newly discovered potential mechanism.

*Allium victorialis* L. (AVL) is a species of the genus Allium (family Alliaceae) and widely spread in most parts of the world. AVL is a kind of medicinal and edible plant, and its stems and leaves are edible. AVL is also used as pickles in soy sauce, wrapped pork, or kimchi, which are loved by many people for their delicious tastes. “Qianjin Yaofang**·**Shi Zhi Juan,” the earliest dietotherapy book in Chinese history written by Sun Si-Miao (the Tang Dynasty, 618–907A.D.), also recorded that AVL could be used to treat liver disease according to the traditional Chinese medicine theory. The ancient traditional Chinese medical books recorded AVL as pungent, slightly warm, and nontoxic and that it could be used to treat body warmth and heat accumulation, clear damp heat, and eliminate miasma, especially in the abdominal digestive system. According to ancient traditional medicine, liver disease is considered to present symptoms of damp heat, toxins, and miasma; therefore, clearing away the heat and removing the dampness is the first choice of treatment. In recent years, AVL has received much attention due to its diverse pharmacological properties, such as anti-obesity, anticancer, antioxidant, hepatoprotective, and so on (Shirataki et al., 2001; Tang et al., 2017). Some studies have found that the extract of AVL could significantly inhibit the formation of tumor nodules in lung tissue and act as a rewarding supplement for cancer prevention and therapy (Kim et al., 2014). Up to now, more than 200 components have been isolated from AVL. The main active components of AVL include volatile oils, flavonoids, steroids, steroidal saponins, carbohydrates, etc. Besides these, plentiful flavonoids isolated from AVL presented functions on regulating neurotransmitters (Woo et al., 2012). In a previous study, the contents of flavonoids in AVL were roughly determined by HPLC analysis by taking rutin, kaempferol, and quercetin as standard references. The authors aimed to explore the improvement effect of AVL on ETOH-induced adipose degeneration in mice, and this study was designed to investigate the effect of AVL on hepatic steatosis and inflammation and reveal the potential role of FXR-mediated by AVL against ALD.

## Materials and Methods

### Plant Material

**FIGURE 1:**
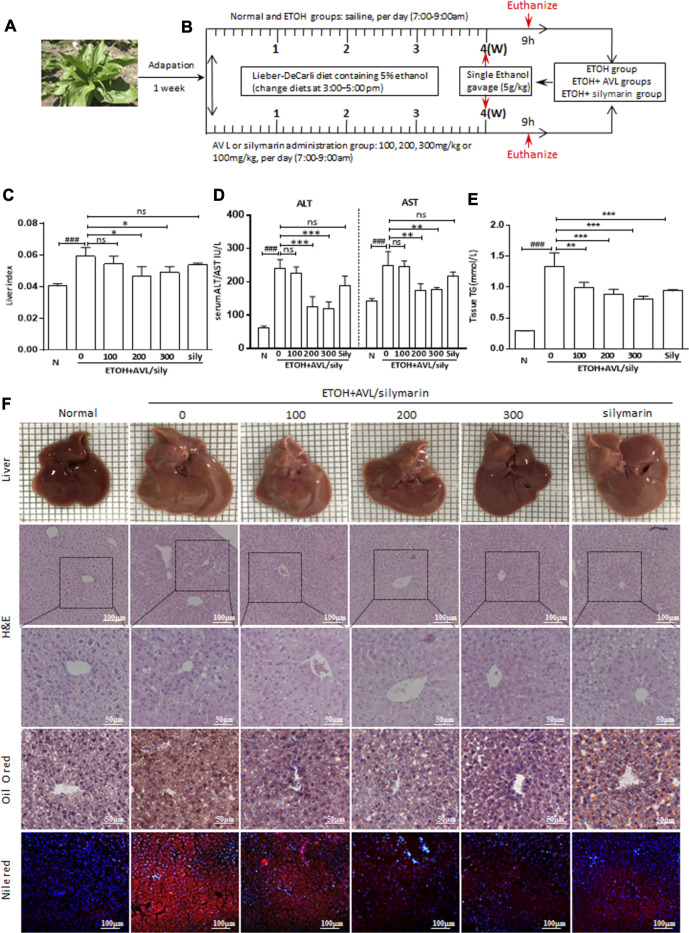
AVL effectively attenuated alcohol-induced fatty liver. **(A)** Picture of AVL. **(B)** Procedures for the animal experiments. **(C)** Liver index levels. **(D)** Serum ALT and AST activities. **(E)** Tissue TG levels. **(F)** Liver appearance picture, H&E stain (×200 and ×400 magnifications), Oil red O stain (×400 magnification), Nile red staining (×200 magnification). ^###^ *p* < 0.001 compared with normal group; *p < 0.05, **p < 0.01, ***p < 0.001 compared with EtOH group; ns, not significant.

The samples of *Allium victorialis* L. (AVL) (Figure 1A) were wild and grown in Hunchun City of Jilin province and were authenticated by Professor Xian Zhang, Agricultural College, Yanbian University, China. A voucher specimen (YBUCP20171212) was deposited in the Herbarium of the Agricultural College, Yanbian University, China.

Fresh AVL (1000 g) was soaked in ethanol for 24 h, followed by reflux extraction for 2 h. After filtration, the filter residue was collected and reflux extraction carried out 3 times. The powder of AVL was obtained by vacuum distillation and drying at 80°C. AVL extracts were analyzed by HPLC in supplement data.

### Reagents

The reagent strips for ALT/AST and TG were purchased from Changchun Huili Biotech Co., Ltd. (Changchun, China). Primary antibodies against GAPDH (ab8245), MPO (ab45977), lipin 1 (ab70138), LXRα (ab41902), lipin 2 (ab176347), CYP2E1 (ab28146), NLRP3 (ab4207), Sirt1 (ab110304), PPARγ (ab19481), SREBP1 (ab3259), F4/80 (ab6640), and Opti-MEM® were obtained from Abcam (Cambridge, MA, United States). Primary antibodies against ASC (sc514414), PPARα (sc9000), LXRβ (sc34341), IL6 (sc28343), IL1R1 (sc393998), and caspase 1 (sc622) were purchased from Santa Cruz Biotechnology Inc. (Santa Cruz, CA, United States). Primary antibodies against FXR (cs4173), p-AMPKα (cs2531), AMPKα (cs2532), p-AMPKβ1/2 (cs4181), AMPKβ1/2 (cs4150), LKB1 (cs3047), p-LKB1 (cs3482), p-ACC (cs11818), and ACC (cs3676) were purchased from Cell Signaling Technology (Beverly, MA, United States). Lipofectamine® 2000 Transfection Reagent was purchased from Thermo Fisher Scientific (Carlsbad, CA, United States). The BCA Protein Assay Kit was obtained from Beyotime (Jiangsu, China). All other chemicals and reagents were obtained from Sigma-Aldrich (Shanghai, China).

### Protocols for the Animal Experiments

Male C57BL/6 mice (body weight, 22–24 g) (SPF, SCXK [J] 2016-0003) purchased from Changchun Yisi Laboratory Animal Technology Co., Ltd. (Jilin, China) were housed under conditions of constant temperature (22 ± 2°C), relative humidity (50–60%), and light (12-h light−dark cycles) for 1 week. The animal experiment was handled in accordance with the Guide for the Care and Use of Laboratory Animals of Yanbian University (Resolution number, 201801022). After a week of acclimatization, all mice were randomly divided into six groups (six mice per group): normal group, EtOH group, EtOH plus AVL (100-, 200-, and 300-mg/kg) groups, and EtOH plus silymarin (100-mg/kg) group. The mice in the normal group were fed control Lieber–DeCarli liquid diet (TP 4030C, Trophic Animal Feed High-Tech Co., Ltd., China), and the mice in the other groups were fed Lieber–DeCarli liquid diet containing ethanol (TP 4030D, Trophic Animal Feed High-Tech Co., Ltd., China). The concentrations of ethanol were from 1 to 4% (v/v) for first 5 days, and then followed by 5% ethanol for 4 weeks. All mice were allowed free access to distilled water during the experiment. EtOH plus AVL or silymarin groups were daily gavaged with AVL (100, 200, and 300 mg/kg) or silymarin (100 mg/kg), and the normal and EtOH groups were daily administrated equal volumes of saline. Except for the normal group, the mice in the other groups were single gavaged with ethanol (5 g/kg) at the end of the fourth week. After the final administration, the mice were sacrificed under anesthesia. Finally, the serum and livers were collected for subsequent experiments. The detailed modeling procedures are described in Figure 1B.

### Cell Culture and Treatment

AML12 and HepG2 cells were generous gifts from Professor Dr. Jung Joon Lee of Korea Research Institute of Bioscience and Biotechnology (Daejeon, Korea). The AML12 cells were incubated in DMEM/F-12 with a mixture of insulin–transferrin–selenium (1%), dexamethasone (40 ng/ml), GlutaMAX (1%), and nonessential amino acids, while HepG2 cells were incubated in DMEM. DMEM contains penicillin (100 U/ml), streptomycin (100 mg/ml), and 10% fetal bovine serum (FBS) under the conditions of 5% CO_2_ at 37°C. For MTT assay, AML12 cells were cultured in 96-well plates at a density of 1 × 10^4^ cells per well and treated with AVL (0–100 μg/ml) or EtOH (0–200 mM). The cells were cultured in 35-mm dishes at a density of 5 × 10^6^ cells per dish and were treated with EtOH (50 mM) with or without AVL.

### Transfection of HepG2 Cells With Plasmid

The transfections of FXR and control plasmid were conducted using the Lipofectamine® 2000 Transfection Reagent according to the manufacturer’s protocols. The cells were cultured in a 24-well plate at a density of 6 × 10^4^ cells per dish, then transfected with Opti-MEM® containing 0.2 μg of FXR plasmid or a negative control and 1.5 μL Lipofectamine® 2000 Transfection Reagent at the confluence of 90–95%. The cells were incubated under the condition of 5% CO_2_ at 37°C for 48 h and then Opti-MEM® substituted with DMEM containing 10% FBS and EtOH (50 mM) with or without AVL (12.5 μg/ml).

### Serum Transaminase and Tissue TG Assay

The serum samples were collected by centrifugation at 3000 rpm and 4°C for 30 min. The liver was homogenized with saline and the homogenate obtained. Serum AST/ALT and tissue TG were measured using Assay kit according to the manufacturer's instructions, which was provided by Changchun Meeting of Biological Co., Ltd. (Changchun, China).

### Immunostaining

Parts of the liver tissues were fixed with 10% formalin solution and embedded in paraffin. Frozen sections were prepared with Tissue-Tek® O.C.T. Compound. After dewaxing hydration, the sections were treated with hematoxylin and eosin (H&E) solution following the manufacturer's instructions. The frozen sections were fixed with the mixture of acetone and methanol or paraformaldehyde, and stained with Oil red O and Nile red stains, also following the manufacturer's instructions. For immunofluorescence (Han et al., 2019), the frozen sections were fixed with the mixture of methanol and acetone, dried at room temperature, washed with PBS, and then blocked with 5% goat serum. The slides were incubated with the primary antibody at 4°C overnight and incubated with Alexa Fluor Goat pAb with rat IgG at room temperature. The nuclei were stained with DAPI and observed under fluorescence microscope.

### Western Blot Analysis

Protein was extracted from liver tissue or cells with RIPA lysis buffer. Equal amounts of protein were separated using 6–15% sodium dodecyl sulfate–polyacrylamide-gel electrophoresis and transferred onto a polyvinylidene fluoride membrane (GE, Freiburg, Germany). The membranes were blocked with skim milk and then incubated with indicated primary antibodies overnight at 4°C, followed with a horseradish peroxidase–conjugated secondary antibody. Finally, the protein bands were visualized with BeyoECL Plus detection reagent (Beyotime, Nanjing, Jiangsu, China) and exposed to an X-ray film. Each band's densitometry was quantified using Bio-Rad Quantity One software.

### Real-Time PCR

Total RNA was isolated from liver tissue or cells using the Eastep Super total RNA Extraction Kit (Promega Biological Products Ltd., Shanghai, China) according to the manufacturer's instructions. Samples of RNA were reverse transcribed into complementary DNA (cDNA). Relative gene expression was assessed by real-time PCR, which was performed on an Agilent Mx3000P QPCR System in a mixture containing Power SYBR® Green PCR Master Mix (Life Technologies, Carlsbad, CA), and specific primers are listed in Table 1. GAPDH was used as a housekeeping gene to quantify relative fold difference, and the relative fold difference was quantified using the comparative threshold cycle (ΔΔCt) method.

**TABLE 1:** Primer sequences used in real-time PCR.

Genes	Sense (5' to 3')	Antisense (3' to 5')
SREBP1	CTTGTGCAGTGCCAGCC	GCC​CAA​TAC​GGC​CAA​ATC​C
FASN	AAA​GTC​CTT​GTC​CAG​GTA​CG	AGG​TCT​TGG​AGA​TGG​CAG​AA
SCD1	TGA​GGC​GAG​CAA​CTG​ACT​AT	GGC​ACC​GTC​TTC​ACC​TTC​T
ACLY	TTG​TGG​ACA​TGC​TCA​GGA​AC	AAG​GTA​GTG​CCC​AAT​GAA​GC
Cpt2	AGT​ATC​TGC​AGC​ACA​GCA​TC	ACT​TCT​GTC​TTC​CTG​AAC​TGG
PPARα	AGG​CTG​TAA​GGG​CTT​CTT​TC	ATT​GTG​TAC​ATC​CCG​ACA​G
ACOX1	CGT​GCA​GCC​AGA​TTG​GTA​G	CGC​CAC​TTC​CTT​GCT​CTT​C
fabp	AAG​TGT​CCG​CAA​TGA​GTT​C	CTT​GAC​GAC​TGC​CTT​GAC​TT
IL18	TGA​CTG​TAG​AGA​TAA​TGC​AC	ATC​ATG​TCC​TGG​GAC​ACT​TC
IL1α	CAG​TGA​AAT​TTG​ACA​TGG​GTG	CAG​GCA​TCT​CCT​TCA​GCA​G
TNF-α	GAG​CAC​TGA​AAG​CAT​GAT​CC	GAG​GGT​TTG​CTA​CAA​CAT​GG
NLRP3	GCC​TAC​AGT​TGG​GTG​AAA​TG	GTC​AGC​TCA​GGC​TTT​TCT​TC
IL1β	TCT​TTG​AAG​AAG​AGC​CCA​TCC	CTA​ATG​GGA​ACG​TCA​CAC​AC
GAPDH	ACC​ACA​GTC​CAT​GCC​ATC​AC	TCC​ACC​ACC​CTG​TTG​CTG​TA

### Statistical Analysis

All data in experiments were expressed as mean ± SD. The comparison between groups was evaluated by GraphPad Prism (GraphPad Software, San Diego, CA, United States). One-way analysis of variance and Tukey's multiple comparison tests were used to perform statistical analyses. Statistically significant differences between groups were defined as *p*-values no more than 0.05.

## Results

### *Allium victorialis* L. Effectively Attenuated Alcohol-Induced Fatty Liver

With the Lieber–DeCarli liquid diet containing 5% (vol/vol) ethanol and single ethanol gavage (5 g/kg), the liver index, serum ALT/AST levels, and hepatic TG levels were significantly increased compared to the normal group, while AVL treatments markedly decreased these alternations compared to the EtOH group (Figures 1C–E).

As shown in Figure 1F, the liver images of the normal mice show a smooth surface, soft texture, and sharp edges. In the EtOH group, the liver tissue of the mice appeared swollen, rough, and tarnished. With AVL or silymarin administration, the liver surface was smoothened and the swelling reduced; however, silymarin administration showed less change than AVL (300 mg/kg).

The H&E analysis showed that the liver in the EtOH group showed massive steatosis compared with that in the normal group, and more fibrous connective tissue in the central region of the hepatic lobules had caused irregular deformation and increased liver injury. All these obvious pathological changes were significantly improved with AVL treatment (Figure 1F). In addition, Oil red O and Nile red staining showed that EtOH could induce the formation of lipid droplets in the liver compared to the normal group; however, lipid droplets in the AVL or silymarin groups were less abundant and much smaller than those in the EtOH group (Figure 1F). These results indicate that AVL showed hepatoprotective effect against liver injury and steatosis induced by EtOH.

### *Allium victorialis* L. Administrations Regulated Lipid-Related Proteins Induced by EtOH

**FIGURE 2:**
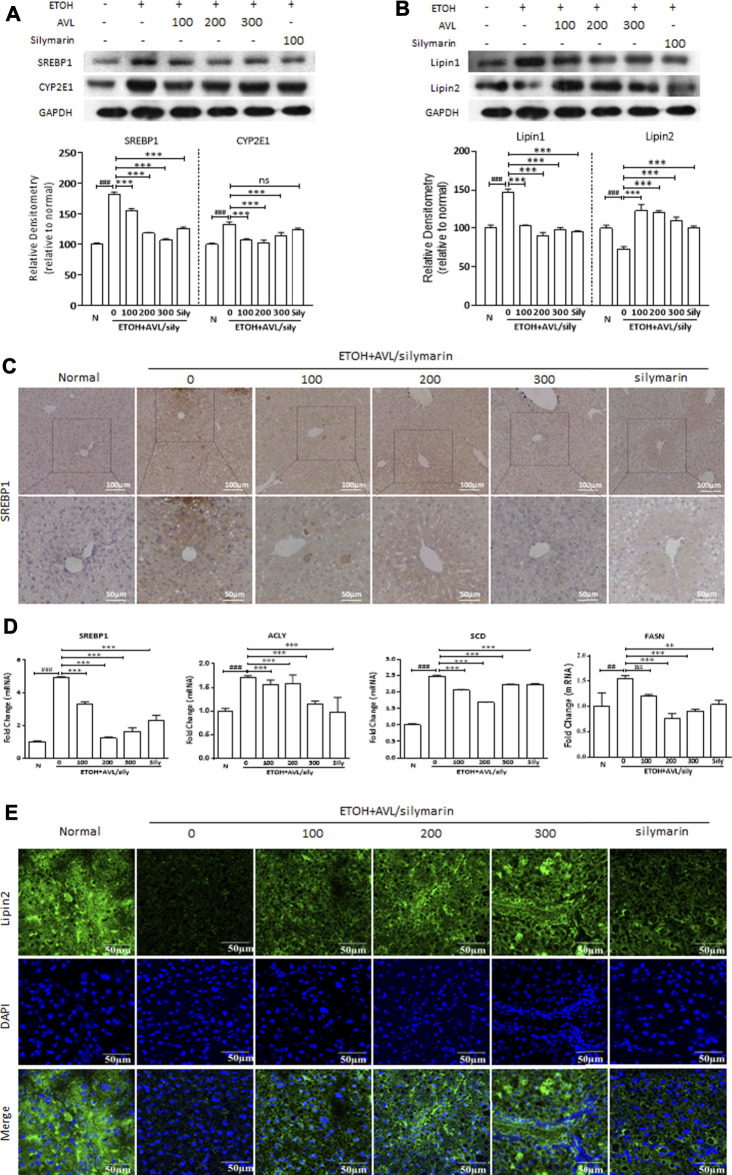
AVL administrations regulated the expression of lipid-related proteins induced by EtOH. **(A)** The effects of AVL on the protein expressions of SREBP1 and CYP2E1. **(B)** The effects of AVL on the protein expressions of lipin 1 and lipin 2. Densitometric values were normalized against GAPDH. The same GAPDH was used in Panels 2A,B and in Figure 3D and Figure 4A. **(C)** Immunohistochemical staining analysis of SREBP1 (×200 and ×400 magnification). **(D)** Representative QPCR analysis for SREBP1, ACLY, SCD, and FASN. **(E)** Immunofluorescence staining analysis of lipin 2 (×600 magnification). Data are presented as mean ± SD (*n* = 3). ##*p* < 0.01, ^###^ *p* < 0.001 compared with normal group; **p < 0.01, ***p < 0.001 compared with EtOH group.

SREBP1 plays a critical role in alcoholic liver disease, and it could regulate the transcription of downstream signaling, such as FASN, SCD, and ACLY. CYP2E1 is a key pathway in the regulation of lipid peroxidation and oxidative stress induced by ethanol. EtOH elevated protein and mRNA levels of SREBP1, the protein level of CYP2E1, and the mRNA levels of FASN, SCD, and ACLY compared to the normal group. AVL treatment could significantly inhibit protein or mRNA expressions of SREBP1, CYP2E1, FASN, SCD, and ACLY compared to the EtOH group (Figures 2A,D), and silymarin showed no obvious regulating in CYP2E1 (Figure 2A). In immunohistochemical staining, AVL or silymarin obviously decreased the positive expressions of SREBP1 (in brown) compared to the ETOH group (Figure 2C).

Both lipin 1 and lipin 2 are the first central regulatory enzymes in the regulation of lipid metabolism in the liver (Song et al., 2018). As is shown in Figure 2B, alcohol intake increased the protein expression of lipin 1 and decreased the protein expression of lipin 2 compared to the normal group, whereas these changes were reversed by AVL treatment compared to the EtOH group (Figure 2B). In Figure 2E, EtOH stimulation obviously inhibited the immunofluorescence expression of lipin 2 (in green) compared to the normal group (Figure 2E), while AVL or silymarin treatment significantly increased the expressions of lipin 2 compared to the EtOH group (Figure 2E). These results suggest that AVL downregulates lipid synthesis and upregulates fatty acid oxidation, and eventually ameliorates hepatic steatosis.

### *Allium victorialis* L. Improved Lipid Accumulation Through Regulation of Farnesoid X Receptor/Liver X Receptor Pathways

**FIGURE 3:**
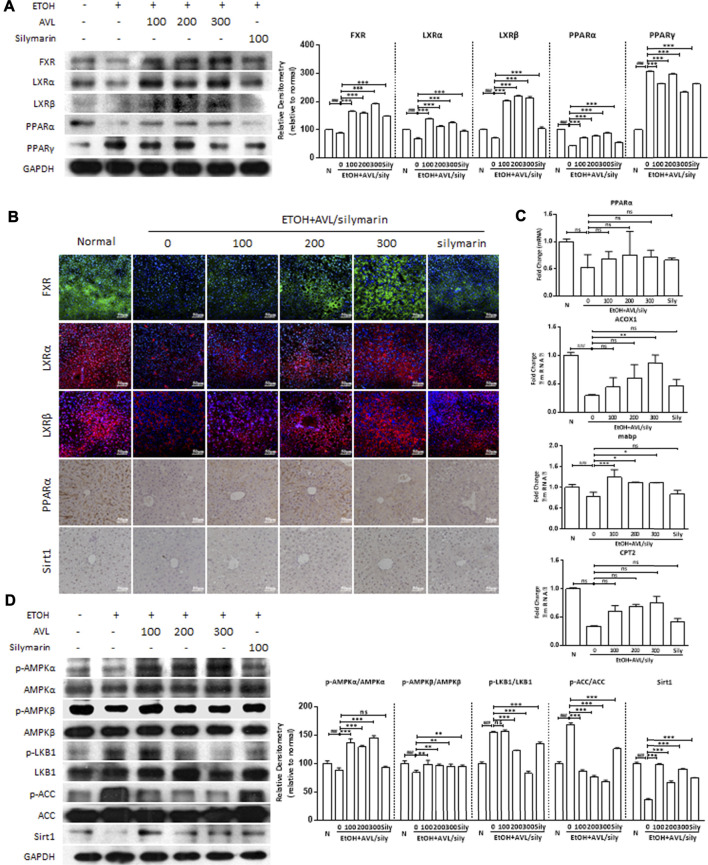
AVL improved lipid accumulation through regulation of FXR/LXR pathways. **(A)** The effects of AVL on the protein expression levels of FXR, LXRα, LXRβ, PPARα, and PPARγ. Densitometric values were normalized against GAPDH. **(B)** Immunofluorescence staining analysis of FXR, LXRα, and LXRβ (×200 magnification), and immunohistochemical staining analysis of PPARα and SIRT1 (×400 magnification). **(C)** Representative QPCR analysis for expressions of mRNA expression of PPARα, ACOX1, mabp, and CPT2. **(D)** The effects of AVL on the protein expression levels of p/t-AMPKα, p/t-AMPKβ, p/t-LKB1, p/t-ACC, and Sirt1. Densitometric values were normalized against GAPDH. The same GAPDH was used in Figures 2A,B, Panel 3D, and Figure 4A. Data are presented as mean ± SD (*n* = 3). ^###^ *p* < 0.001 compared with normal group; *p < 0.05, **p < 0.01, ***p < 0.001 compared with EtOH group; ns, not significant.

FXR and liver X receptors (LXRs), which belong to the NRs supergene family, can regulate the lipid metabolism gene in metabolic diseases. As shown in Figure 3A, ethanol significantly decreased the protein expressions of FXR, LXRα, and LXRβ compared to the normal group, whereas AVL or silymarin treatments significantly increased the expressions of FXR, LXRα, and LXRβ compared to the EtOH group. Consistent with these changes, the results of the immunofluorescence staining showed that positive expressions of LXRα, LXRβ (in red), and FXR (in green) were significantly decreased in the EtOH group than in the normal group, AVL treatments significantly enhanced these expressions compared to the EtOH group, and silymarin had little effect on the expressions of these proteins (Figure 3B). These results indicate that AVL regulated lipid metabolism by FXR and LXRs activation.

PPARα and PPARγ, members of the ligand-activated NRs transcription factor superfamily, are involved in lipogenesis, and in energy and glucose homeostasis. In the EtOH group, the protein expression of PPARα was deceased and that of PPARγ increased compared to the normal group, whereas AVL treatments significantly increased the expression of PPARα and decreased the expression of PPARγ compared to the EtOH group (Figure 3A). The regulation of AVL on PPARα expression was also supported by immunohistochemistry staining, which showed positive expression (in brown) (Figure 3B). Moreover, alcohol intake decreased mRNA expressions of the PPARα-regulated genes—ACOX1, mabp, and CPT2, while AVL administrations obviously ameliorated these changes caused by EtOH. These results demonstrate that AVL regulated PPARα-mediated fatty acid oxidation (Figure 3C). In addition, AVL significantly regulated AMPKα/β, LKB1, and ACC phosphorylation and Sirt1 compared to the ETOH group (Figure 3D). The effect of AVL on Sirt1 was also verified by the immunohistochemistry staining (Figure 3B). Thus, AVL regulated AMPK/LKB1/ACC and Sirt1 signaling pathway against ALD.

### *Allium victorialis* L. Ameliorated Ethanol-Induced Hepatic Inflammation

**FIGURE 4:**
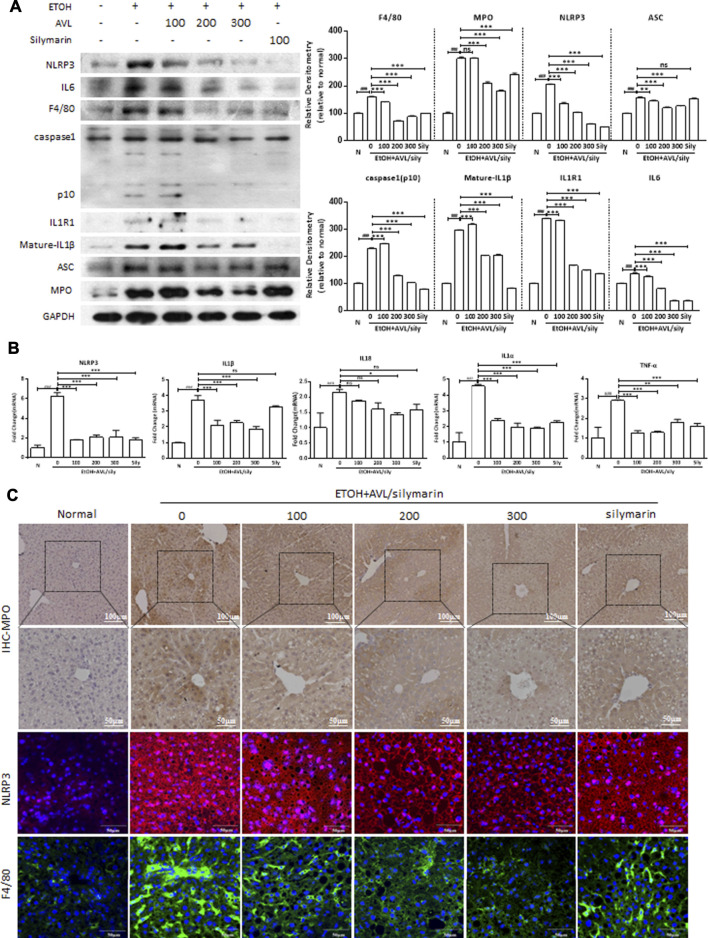
AVL ameliorated ethanol-induced hepatic inflammation. **(A)** The effects of AVL on the protein expressions of NLRP3, IL6, F4/80, caspase1, IL1R1, IL1β, ASC, and MPO. Densitometric values were normalized against GAPDH. The same GAPDH was used in Figure 2A,B, Figure 3D, and Panel 4A. **(B)** Representative QPCR analysis for NLRP3, ASC, IL6, caspase1-p10, IL1R1, and mature-IL1β. **(C)** Immunohistochemical staining analysis of MPO (×200 and ×400 magnification), and immunofluorescence staining analysis of NLRP3 and F4/80 (×600 magnification). Data are presented as mean ± SD (*n* = 3). ^###^ *p* < 0.001 compared with normal group; *p < 0.05, **p < 0.01, ***p < 0.001 compared with EtOH group; ns, not significant.

Inflammation plays a key role in the development of hepatic fibrosis. Inflammasomes are large intracellular multiprotein complexes involved in the development of inflammatory disorders, and NLRP3 is a danger-signal sensor to a regulatory node of inflammatory diseases. The protein expression of NLRP3 significantly increased in the EtOH group than in the normal group (Figure 4A). EtOH resulted in a marked inflammatory response in the liver as evidenced by increased expression levels of NLRP3 and ASC, which promoted the inflammatory response with releases of inflammatory cytokines, including IL6, caspase1-p10, IL1R1, and IL1β. Caspase-1 is a protease associated with inflammatory reaction and produces mature IL1β and IL18. Thus, we found AVL and silymarin administrations significantly decreased the expressions of NLRP3, ASC, IL6, caspase1, IL1R1, and IL1β. However, silymarin showed no significant decrease of ASC compared to the EtOH group (Figure 4A). The mRNA expressions of NLRP3, IL1β, IL18, IL1α, and TNF-α were significantly decreased by AVL (Figure 4B). Immunofluorescence staining also indicated that AVL significantly decreased the expressions of MPO, NLRP3, and F4/80 compared to the EtOH group (Figure 4C). These results demonstrated that AVL inhibits the release of inflammatory factors and the related inflammatory response, and further ameliorated ethanol-induced hepatic inflammation.

### *Allium victorialis* L. Improved Lipid Accumulation and Inflammation *in vitro*

**FIGURE 5:**
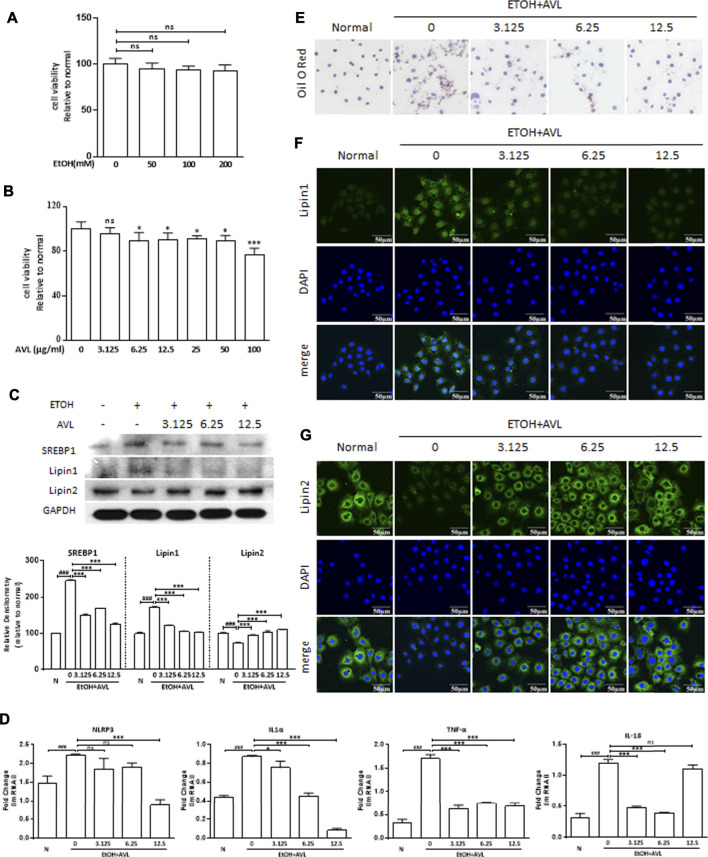
AVL improved lipid accumulation and inflammation *in vitro*. **(A)** MTT assay on cell viability of AML12 with EtOH treatment. **(B)** MTT assay on cell viability of AML12 with AVL treatment. **(C)** The effects of AVL on the protein expressions of SREBP1, lipin 1, and lipin 2. Densitometric values were normalized against GAPDH. The same GAPDH was used in Panel 5C and Figure 6A. **(D)** Representative QPCR analysis for NLRP3, IL1α, TNF-α, and IL18. **(E)** Oil red O staining. **(F)** Immunofluorescence staining analysis for lipin 1. **(G)** Immunofluorescence staining analysis for lipin 2. Data are presented as mean ± SD (*n* = 3). ^###^ *p* < 0.001 compared with normal group; *p < 0.05, **p < 0.01, ***p < 0.001 compared with EtOH group; ns, not significant.

EtOH (50, 100, and 200 mM) did not significantly reduce the cell viability of AML12 compared with the negative control (Figures 5A,B). AVL (6.25–100 µM) significantly reduced the cell viability of AML12 compared with the negative control without AVL (Figure 5B). Consequently, ETOH (50 mM) and AVL (3.125, 6.25, and 12.5 µM) were chosen in the subsequent experiments.

AVL could significantly regulate protein expressions of SREBP1 and lipin 1/2 compared to the EtOH group (Figure 5C), which was supported by the immunofluorescence staining of lipin 1/2 (Figures 5F,G). In Oil red O staining, AVL obviously decreased the lipid droplets induced by EtOH (Figure 5E). AVL treatments could inhibit NLRP3, IL1α, TNF-α, and IL18 at the gene level (Figure 5D). These results demonstrate that AVL could inhibit lipid droplets by regulating the expression of SREBP1, lipin 1/2, and inflammation *in vitro*.

### Farnesoid X Receptor Is Necessary for *Allium victorialis* L. to Ameliorated EtOH-Induced Liver Lipid Deposition

**FIGURE 6:**
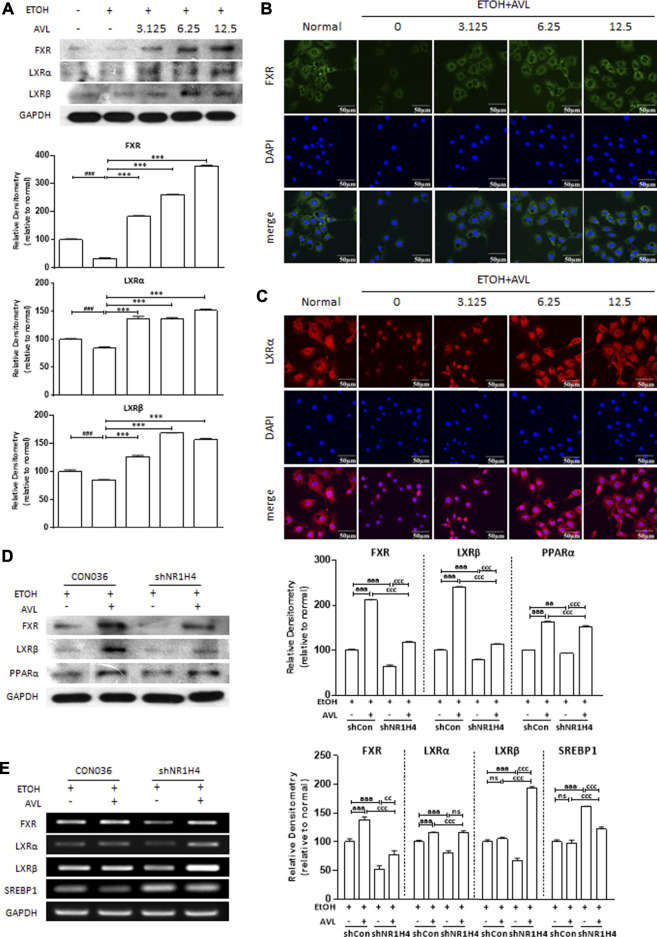
FXR is necessary for AVL to ameliorated EtOH-induced liver lipid deposition. **(A)** The effects of AVL on the protein expressions of FXR, LXRα, and LXRβ in AML12 cells. Densitometric values were normalized against GAPDH. The same GAPDH was used in Figure 5C and Panel 6A. **(B)** Immunofluorescence staining analysis for FXR in AML12 cells. **(C)** Immunofluorescence staining analysis for LXRα in AML12 cells. Densitometric values were normalized against GAPDH. Data are presented as mean ± SD (*n* = 3). ^##^ *p* < 0.001, ^###^ *p* < 0.001 compared with the normal group; ***p < 0.001 compared with the ETOH group. **(D)** The effects of AVL on the protein expressions of FXR, LXRα, LXRβ, and PPARα undergoing shFXR. **(E)** Representative real-time PCR analysis for expressions of FXR, LXRα, LXRβ, and SREBP1 undergoing shFXR. Densitometric values were normalized against GAPDH. ^aaa^ *p* < 0.001 CON036 treatment vs CON036-AVL group, ^cc^ *p* < 0.01, ^ccc^ *p* < 0.001 CON036-AVL vs shRNA (FXR)-AVL group, shRNA (FXR) vs shRNA (FXR)-AVL group; ns, not significant.

The LXRs and FXR could regulate the lipid metabolism gene in metabolic and digestive diseases. As we expected, AVL treatments could reverse the reductions of FXR, LXRα, and LXRβ in AML12 cells stimulated with EtOH (Figure 6A), which were further verified by immunofluorescent staining of FXR and LXRα (Figures 6B,C).

To further confirm that AVL ameliorates alcoholic fatty liver by activating FXR mediation on lipid accumulation, HepG-2 cells were transfected with FXR (NR1H4) shRNA and then treated with EtOH, and with or without AVL. The FXR deficiency was appropriately achieved by FXR shRNA (Figure 6D,E). EtOH-mediated decrease of LXRα and LXRβ was strengthened by FXR shRNA, which was also attenuated by AVL interference (Figures 6D,E). FXR deficiency also resulted in the decreasing of PPARα (Figure 6D), and the increase of SREBP1 (Figure 6E). These results suggest that FXR is necessary for AVL to ameliorated EtOH-induced liver lipid deposition, and FXR activation might be the potential therapeutic target for AVL against ALD.

## Discussion

Excessive alcohol consumption is responsible for the development of ALD, with a spectrum comprising alcoholic fatty liver, alcoholic steatohepatitis, and so on. The present study found that AVL could attenuate ALD induced by Lieber–DeCarli liquid diet containing ethanol. The mouse model induced by Lieber–DeCarli ethanol liquid diet plus single-binge ethanol feeding synergistically induces liver injury, inflammation, and fatty liver, which mimics acute-on-chronic alcoholic liver injury in patients (Bertola et al., 2013). AVL is a medical and edible plant, which is the guarantee for the safety of AVL usage. Our previous study also showed that AVL is safe for use alone in treatment and caused no liver histopathological changes. AVL could decrease liver index, serum transaminase, and TG accumulation, which has been further verified by histopathological examination. AVL also could ameliorate metabolic disorders and inflammation by inhibiting liver lipid deposition and inflammation factors caused by alcohol. In EtOH-induced metabolic dysregulation, it was found that FXR played an important role during AVL mediation against ALD, and FXR was necessary for AVL to regulate alcoholic steatosis and meta-inflammation.

Studies have found that FXR is a key bile acid–activated receptor that plays a critical role in the regulation of lipid and glucose metabolisms, anti-inflammation, cholestasis, and so on (Tung and Carithers, 1999; Ferrell et al., 2019). The action of activated FXR on the regulation of lipogenesis can be attributed to the downregulation of TG levels. Activated FXR can downregulate the expressions of SREBP1C and its downstream target genes, such as FAS, SCD-1, and ACC, which are related to fatty acid synthesis and TGs by inducing the expression of SHP (Watanabe et al., 2004; Song et al., 2020). Consistent with previous studies, the current results indicate that AVL can activate FXR to regulate lipid deposition, which has been verified by the decrease of SREBP1 and its target genes, and of PPARα and its target genes. The silencing of the FXR gene *in vitro* further indicates that FXR is necessary for AVL in regulating alcohol-induced lipid accumulation and inflammation. Our study provides new insights into the mechanism by which FXR controls metabolic disorders and inflammation in ALD.

Moreover, the increase of SREBP1 may relate to AMPK phosphorylation. AMPK plays a critical role in regulating fatty acid oxidation pathways and inhibiting lipid synthesis. Alcohol can inhibit AMPK phosphorylation, and increase the expression of SREBP1 and suppressed adenylyl cyclase activity, finally contributing to hepatic steatosis (Subauste and Burant, 2007; Yao et al., 2017a; Yao et al., 2017b). Studies have shown that both Sirt1 and AMPK are two key factors in controlling lipid metabolism (Zhu et al., 2016). Activated Sirt1 could promote AMPK phosphorylation, and it also can be regulated by phosphorylation AMPK on the contrary, while loss of Sirt1 could increase hepatic steatosis and inflammation in mice (Choi et al., 2015; Li et al., 2015). In addition, AMPK phosphorylation can be activated by FXR and mediate oxidative stress (Zhang et al., 2017). These results may suggest that AVL can activate the Sirt1–AMPK signaling pathway via activation of FXR to further inhibit lipid accumulation.

Besides FXR, NRs also include PPARs, LXRs, and PXP, which are associated with various pathologies, such as cholestasis, inflammation, hepatic steatosis, fibrosis, and cancer (Tardelli et al., 2018). Among these NRs, LXRs play an important role in the metabolism of lipids, bile acids, and carbohydrates, and PPARγ with LXR could modulate macrophage activation by regulating several anti-inflammatory responses (Han et al., 2021). In the present study, the silencing of the FXR gene could decrease expressions of PPARα and LXRβ, which might lead to the release of inflammatory cytokines and the increase of SREBP1 expression, which further prove that AVL ameliorates ALD by targeting FXR activation.

Ethanol can regulate the expression of lipin 1/2 by regulating AMPK and SREBP1 signaling pathways (Bi et al., 2015). Lipin 1 can promote the synthesis of TGs and the oxidation of fatty acids during lipid metabolism (You et al., 2017). Like lipin 1, lipin 2 also plays a critical role in lipid metabolism, and it can regulate NLRP3 inflammasome by activating the P2X7 receptor, which in turn regulates inflammatory responses (Lordén et al., 2017). As expected, alcohol can upregulate the expression of lipin 1 and downregulate lipin 2, which were related to lipid metabolism and inflammatory, while AVL can reverse the changes of lipin 1 and lipin 2 induced by alcohol and further decrease lipid accumulation and the release of inflammatory cytokines. All these results demonstrate that AVL can ameliorate ethanol-induced liver injury by regulating energy metabolism and inflammation.

In conclusion, the findings of this study suggest that AVL would ameliorate alcoholic steatohepatitis, lipid deposition, and inflammation in ALD by targeting FXR activation, and further present that AVL targeting FXR might be an attractive candidate or strategy for ALD treatment. As a medicinal food homologous plant, AVL is worth including in product development for potent anti-alcohol related diseases and can be widely applied in the health industry. However, further studies may need to focus on the effective parts or chemical components isolated from AVL to improve its efficacy and targeted regulation accuracy.

## Data Availability

The raw data supporting the conclusion of this article will be made available by the authors, without undue reservation.

## Abbreviations

ALD, alcoholic liver disease; AMPK, adenosine 5‘-monophosphate (AMP)-activated protein kinase; CYP2E1, cytochrome P4502E1; FAS, fatty acid synthase; FXR, farnesoid X receptor; LXRs, liver X receptors; NLRP3, NLR family pyrin domain containing 3; NRs, nuclear hormone receptors; PPARα, peroxisome proliferators-activated receptor α; RXRα, retinoid X receptor α; SCD-1, stearoyl-coenzyme A desaturase 1; SIRT1, NAD-dependent deacetylase Sirtuis; SREBP1, sterol-regulatory element binding protein 1.
